# Genetic susceptibility for chronic bronchitis in chronic obstructive pulmonary disease

**DOI:** 10.1186/s12931-014-0113-2

**Published:** 2014-09-21

**Authors:** Jin Hwa Lee, Michael H Cho, Craig P Hersh, Merry-Lynn N McDonald, James D Crapo, Per S Bakke, Amund Gulsvik, Alejandro P Comellas, Christine H Wendt, David A Lomas, Victor Kim, Edwin K Silverman, on behalf of the COPDGene and ECLIPSE Investigators

**Affiliations:** Channing Division of Network Medicine, Brigham and Women’s Hospital, Boston, MA USA; Division of Pulmonary and Critical Care Medicine, Department of Internal Medicine, School of Medicine, Ewha Womans University, Seoul, South Korea; Division of Pulmonary and Critical Care Medicine, Brigham and Women’s Hospital, Boston, MA USA; National Jewish Health, Denver, CO USA; Department of Clinical Science, University of Bergen, Bergen, Norway; Department of Thoracic Medicine, Haukeland University Hospital, Bergen, Norway; Department of Pulmonary Medicine, University of Iowa, Iowa City, IA USA; Department of Medicine, VA Medical Center, University of Minnesota, Minneapolis, MN USA; Wolfson Institute for Biomedical Research, University College London, London, UK; Temple University School of Medicine, Philadelphia, PA USA

**Keywords:** Pulmonary disease, Chronic obstructive, Chronic bronchitis, Genome-wide association study

## Abstract

**Background:**

Chronic bronchitis (CB) is one of the classic phenotypes of COPD. The aims of our study were to investigate genetic variants associated with COPD subjects with CB relative to smokers with normal spirometry, and to assess for genetic differences between subjects with CB and without CB within the COPD population.

**Methods:**

We analyzed data from current and former smokers from three cohorts: the COPDGene Study; GenKOLS (Bergen, Norway); and the Evaluation of COPD Longitudinally to Identify Predictive Surrogate Endpoints (ECLIPSE). CB was defined as having a cough productive of phlegm on most days for at least 3 consecutive months per year for at least 2 consecutive years. CB COPD cases were defined as having both CB and at least moderate COPD based on spirometry. Our primary analysis used smokers with normal spirometry as controls; secondary analysis was performed using COPD subjects without CB as controls. Genotyping was performed on Illumina platforms; results were summarized using fixed-effect meta-analysis.

**Results:**

For CB COPD relative to smoking controls, we identified a new genome-wide significant locus on chromosome 11p15.5 (rs34391416, OR = 1.93, *P* = 4.99 × 10^-8^) as well as significant associations of known COPD SNPs within *FAM13A*. In addition, a GWAS of CB relative to those without CB within COPD subjects showed suggestive evidence for association on 1q23.3 (rs114931935, OR = 1.88, *P* = 4.99 × 10^-7^).

**Conclusions:**

We found genome-wide significant associations with CB COPD on 4q22.1 (*FAM13A*) and 11p15.5 (*EFCAB4A*, *CHID1* and *AP2A2*), and a locus associated with CB within COPD subjects on 1q23.3 (*RPL31P11* and *ATF6*). This study provides further evidence that genetic variants may contribute to phenotypic heterogeneity of COPD.

**Trial registration:**

ClinicalTrials.gov NCT00608764, NCT00292552

**Electronic supplementary material:**

The online version of this article (doi:10.1186/s12931-014-0113-2) contains supplementary material, which is available to authorized users.

## Background

COPD, a leading cause of morbidity and mortality, is characterized by persistent airflow limitation and phenotypic heterogeneity. While cigarette smoking is a major risk factor for COPD, the response to cigarette smoke is highly variable [1]. Chronic bronchitis (CB) and emphysema represent two classic phenotypes of COPD [2]. However, CB, which is defined clinically by chronic cough and phlegm, can occur in the absence of COPD [3]. Some studies have suggested that CB and emphysema have different genetic determinants [4,5]. CB has been reported to be associated with frequent respiratory exacerbations, increased respiratory symptoms, poor quality of life, and even increased mortality [6-8].

Although candidate gene testing and linkage analysis have been used to search for CB-related genetic determinants in selected populations [9,10] and recently a genome-wide association meta-analysis has reported genetic variants associated with chronic mucus hypersecretion mainly in subjects from the general population [11], genome-wide association studies (GWAS) of CB within COPD subjects have not been reported. Our primary hypothesis was that genetic variants would be associated with COPD-related CB. We also hypothesized that genetic heterogeneity exists according to the presence or absence of CB within COPD subjects. We addressed these hypotheses by comparing COPD subjects with CB to smokers with normal spirometry and to COPD subjects without CB as control groups.

## Methods

### Study cohorts

Subjects were current and former smokers from three studies: the non-Hispanic whites (NHWs) from the COPDGene Study (NCT00608764 at, https://clinicaltrials.gov); GenKOLS (Bergen, Norway); and the Evaluation of COPD Longitudinally to Identify Predictive Surrogate Endpoints (ECLIPSE, NCT00292552 at, https://clinicaltrials.gov). All subjects had self-described European white ancestry. The design and procedures for each participating study have been previously described [12-14]. For supplementary analysis, the African Americans (AAs) of the COPDGene Study were included. Institutional review board approval was obtained at each participating clinical center; all subjects provided written informed consent. This study was approved by the Partners HealthCare Institutional Review Board (COPDGene, 2007P000554; GenKOLS, 2009P000790; ECLIPSE, 2005P002467).

### Variable definitions

**Figure 1:**
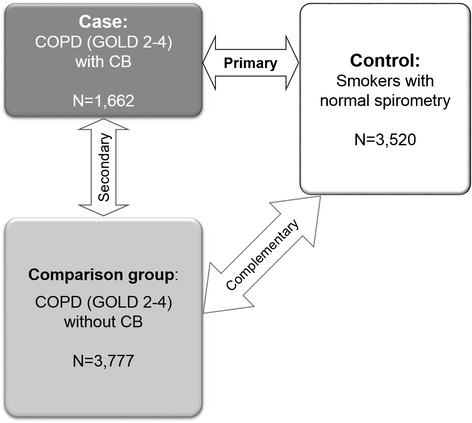
**Genome-wide association study design for chronic bronchitis.** Definition of abbreviations: CB = chronic bronchitis; COPD = chronic obstructive pulmonary disease; GOLD = Global initiative for chronic Obstructive Lung Disease. GOLD 2-4 was defined as having a post-bronchodilator FEV1/FVC < 0.7 and FEV1 < 80% predicted. Normal spirometry was defined as having a post-bronchodilator FEV1/FVC ≥ 0.7 and FEV1 ≥ 80% predicted.

CB was defined as chronic productive cough for 3 months in each of 2 successive years [15]. CB COPD cases were defined as having both CB and COPD of at least spirometry grade 2 (post-bronchodilator FEV_1_/FVC <0.7 and FEV_1_ < 80% predicted), defined by the Global initiative for chronic Obstructive Lung Disease (GOLD 2-4) [16]. For CB COPD cases, primary analysis used current or former smokers with normal spirometry (post-bronchodilator FEV_1_/FVC ≥0.7 and FEV_1_ ≥ 80% predicted) as a control group. A secondary analysis was performed using COPD subjects without CB as controls to explore genetic heterogeneity within COPD subjects. Additionally, we performed GWAS of COPD subjects without CB relative to smoking controls for comparison to our results in COPD CB subjects (Figure 1). Additional variable definitions for complementary analyses are available in an online supplement.

### Genotyping quality control and imputation

Genotyping was performed using Illumina platforms [HumanOmniExpress for the COPDGene cohort, the HumanHap 550 (V1, V3, and Duo) for the GenKOLS cohort, and HumanHap 550V3for the ECLIPSE cohort; Illumina, Inc., San Diego, CA]. Genotype imputation on the COPDGene cohorts was performed using MaCH [17] and minimac [18] using 1000 Genomes [19] Phase I v3 European (EUR) reference panels or cosmopolitan reference panels for NHWs and AAs, respectively. Details on genotyping quality control and imputation for the GenKOLS and ECLIPSE cohorts have been previously described [5,14,20-23]. If variants passed genotyping or imputation quality control in all three cohorts, they were included for analysis.

### Statistical analysis

Logistic regression analysis of SNPs under an additive model of inheritance with case-control status as the outcome was performed in each cohort with adjustment for age, gender, pack-years of cigarette smoking and genetic ancestry-based principal components using PLINK 1.07 [24], as previously described [21-23]. Imputed genotypes were analyzed in a similar manner, using SNP dosage data in PLINK 1.07 [24]. We performed fixed-effects meta-analysis [25] using METAL (version 2011-3-25) [26] and R 2.15.1 (www.r-project.org) with the meta-package. Heterogeneity was assessed by calculating both *I*^*2*^ [27] and *P* values for Cochran’s *Q.* In genomic regions with evidence of genetic heterogeneity, we also used a modified random-effects model optimized to detect associations under heterogeneity since the fixed effects model is based on inverse-variance-weighted effect size [28]. Genomic inflation factors [29] were calculated using GenABEL [30]. We used LocusZoom [31] to generate regional association plots, using the 1000 Genomes EUR reference data to calculate linkage disequilibrium (LD).

We used permutation testing [23] to assess differences in ORs of previous known genome-wide significant SNPs between two meta-analyses.

## Results

### GWAS of CB COPD relative to smokers with normal spirometry

Baseline characteristics of each of the three primary cohorts are summarized in Table 1.

**Table 1:** **Baseline characteristics of COPD subjects with chronic bronchitis and smokers with normal spirometry as a control group**

	**COPDGene NHWs**		**GenKOLS**		**ECLIPSE**	
	**COPD with CB**	**Controls**	**COPD with CB**	**Controls**	**COPD with CB**	**Controls**
n	844	2,534	311	808	507	178
Age, years	62.8 (8.3)	59.5 (8.7)	65.3 (10.0)	55.6 (9.7)	62.3 (7.7)	57.5 (9.4)
Pack-years	59.2 (28.6)	37.8 (20.3)	33.9 (20.2)	19.7 (13.6)	51.6 (30.2)	32.1 (24.8)
Current smoker (%)	53.3	39.6	54.0	41.2	51.4	40.1
FEV_1_,% predicted	48.6 (17.4)	96.8 (11.0)	46.9 (16.8)	94.9 (9.2)	46.3 (15.3)	107.9 (13.7)
Sex (% male)	61.8	49.3	64.6	50.1	75.7	57.9

Data are presented as mean (SD) or percentage, as appropriate.

Definition of abbreviations: *CB* chronic bronchitis, *NHW* non-Hispanic white.

**Figure 2:**
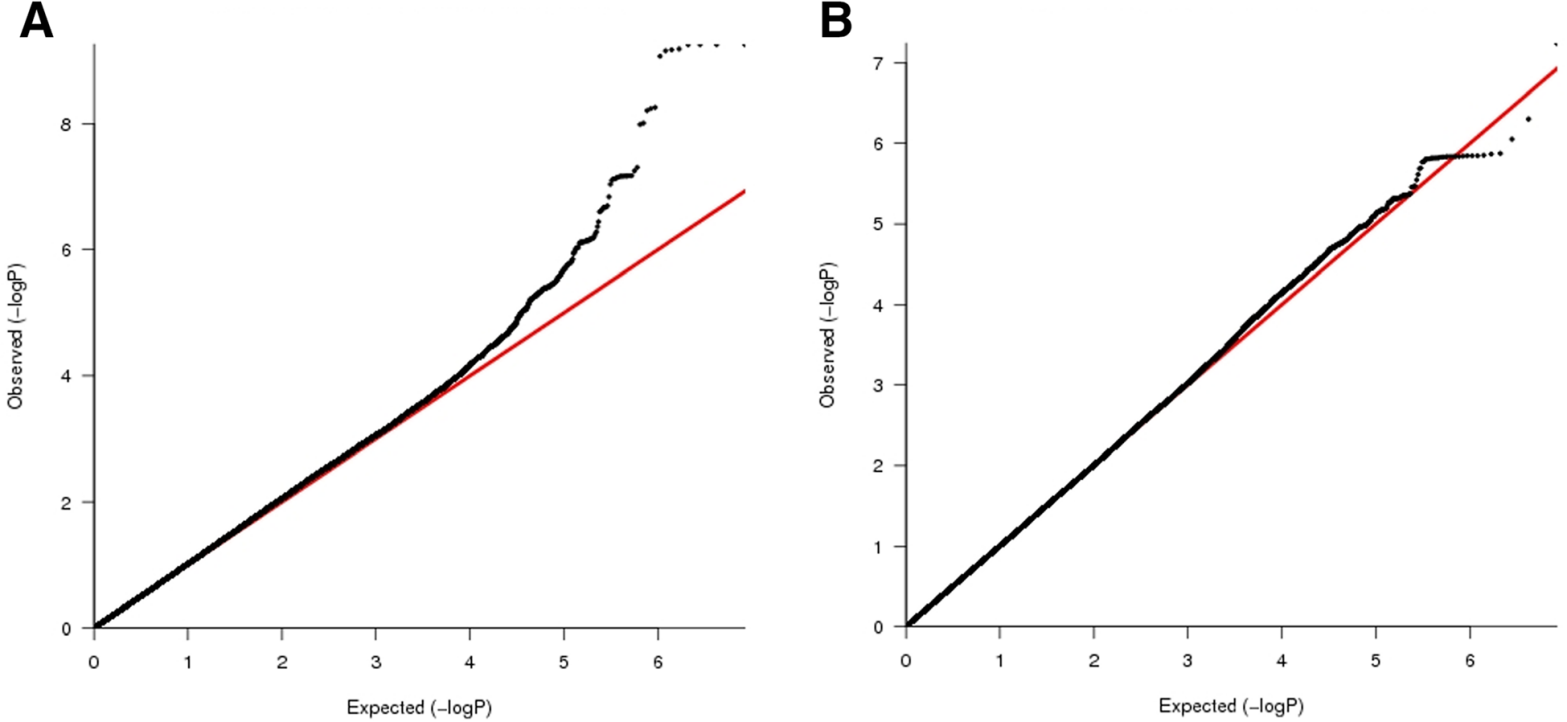
**The quantile–quantile plots for the three-cohort meta-analysis including 1000 Genomes project imputed data for (A) COPD subjects with chronic bronchitis (CB)** ***versus*** **smoking controls and (B) CB** ***versus*** **no CB within COPD subjects, after adjustment for age, sex, pack-years of cigarette smoking and genetic ancestry using principal components.**

**Figure 3:**
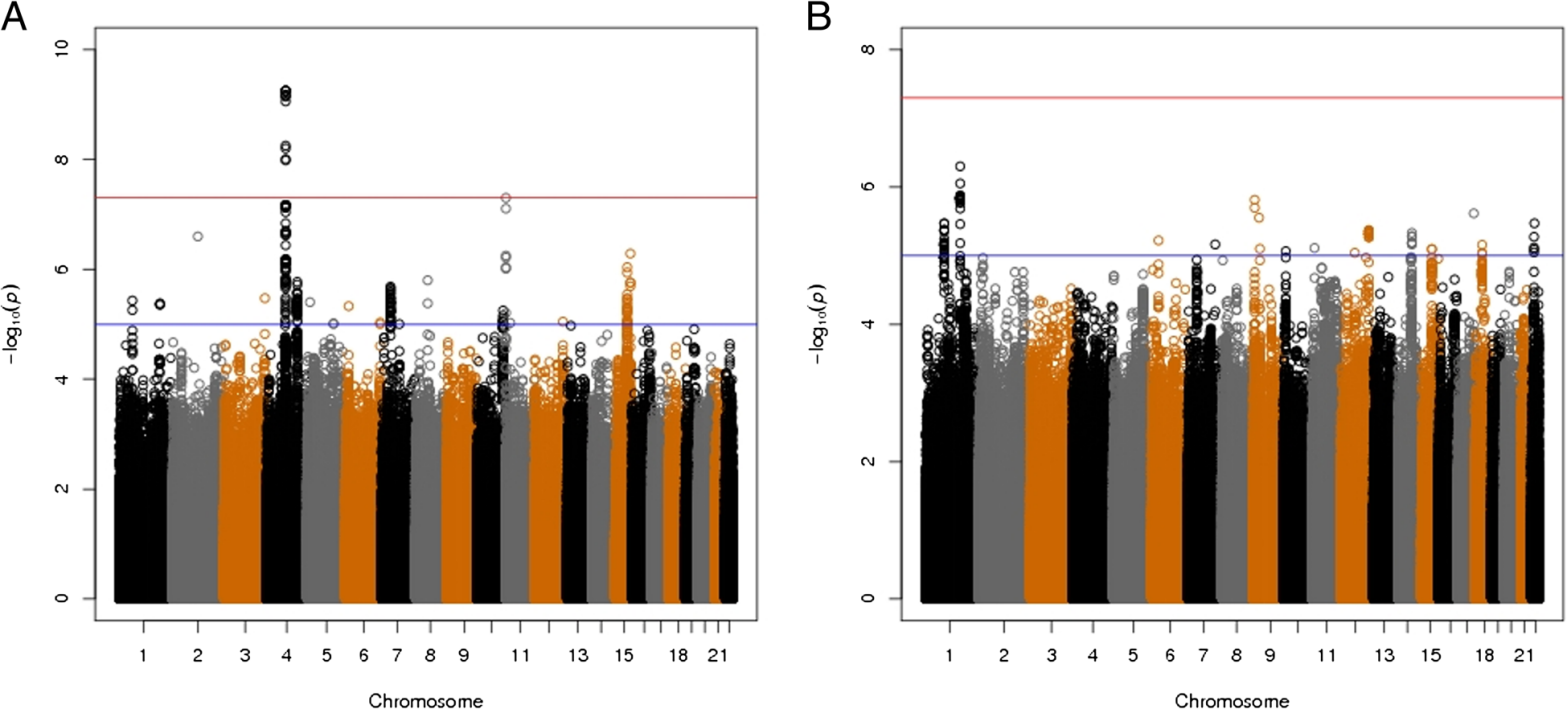
**Manhattan plots of –log**_**10**_ ***P*** **values for meta-analysis of three cohorts for (A) COPD subjects with chronic bronchitis (CB)** ***versus*** **smoking controls and (B) CB** ***versus*** **no CB within COPD subjects, after adjustment for age, sex, pack-years of cigarette smoking and genetic ancestry using principal components.**

**Figure 4:**
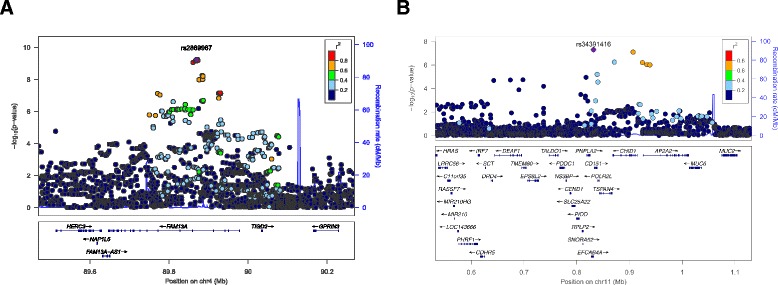
**Local association plots for significant loci in the meta-analysis of cases with chronic bronchitis and COPD versus smoking control subjects in COPDGene non-Hispanic whites, GenKOLS, and ECLIPSE. A**. rs2869967 on chromosome 4q22.1. **B**. rs34391416 on 11p15. The x-axis is chromosomal position, and the y-axis shows the –log10 P value. The most significant SNP at each locus is labeled in purple, with other SNPs colored by degree of linkage disequilibrium (r2). Plots were created using LocusZoom.

For the primary analysis of CB COPD relative to smokers with normal spirometry, the combined GWAS of three cohorts included 1,662 CB COPD cases and 3,520 controls. The quantile-quantile (Q-Q) plot showed no evidence of significant residual population stratification (Figure 2A; lambda = 1.03). Figure 3A shows a genome-wide significant association within the previously reported COPD susceptibility genome-wide significant region on chromosome 4q22.1 in *FAM13A* and a second genome-wide significant association in a novel region on 11p15.5. The results for the most significant SNPs at each of these loci are listed in Table 2. Figure 4 displays the regional association plots for these two regions. The top 12 SNPs in the meta-analysis were located on 4q22.1 (*FAM13A*) and either identical to, or in strong LD (r^2^ ≥ 0.97) with, the top SNPs previously described in GWASs of pulmonary function [32,33] and COPD [21-23].

**Table 2:** **Top results of the meta-analysis for COPD subjects with chronic bronchitis** ***versus*** **smokers with normal spirometry in COPDGene non-Hispanic white, GenKOLS, and ECLIPSE studies** ^*^

**Locus**	**Nearest gene**	**SNP**	**Risk allele**	**FRQ**	**COPDGene NHWs**		**GenKOLS**		**ECLIPSE**		**Overall**			
					**OR (95% CI)**	***P***	**OR (95% CI)**	***P***	**OR (95 CI)**	***P***	**OR (95% CI)**	***P***	***I*** ^***2***^	***Q***
**4q22**	*FAM13A*	rs2869967	C	0.41	1.33 (1.17-1.51)	9.92 × 10^-6^	1.56 (1.25-1.95)	8.08 × 10^-5^	1.40 (1.04-1.88)	2.66x10^-2^	1.38 (1.25-1.53)	5.61 × 10^-10^	0	0.46
**4q22**	*FAM13A*	rs2045517	T	0.40	1.33^†^ (1.17-1.51)	9.87 × 10^-6^	1.56^†^ (1.25-1.95)	7.88 × 10^-5^	1.40^†^ (1.04-1.88)	2.67 × 10^-2^	1.38 (1.25-1.53)	5.63 × 10^-10^	0	0.46
**4q22**	*FAM13A*	rs7671167	T	0.51	1.32 (1.16-1.49)	1.69 × 10^-5^	1.44 (1.15-1.80)	1.27 × 10^-3^	1.41 (1.06-1.87)	1.73 × 10^-2^	1.35 (1.22-1.50)	5.58 × 10^-9^	0	0.75
**4q22**	*FAM13A*	rs2904259	C	0.50	1.31^†^ (1.16-1.49)	1.85 × 10^-5^	1.44^†^ (1.15-1.80)	1.33 × 10^-3^	1.41^†^ (1.06-1.87)	1.73 × 10^-2^	1.35 (1.22-1.49)	6.25 × 10^-9^	0	0.75
**11p15**	*EFCAB4A*	rs34391416	A	0.05	2.38 (1.81-3.13)	6.11 × 10^-10^	1.21^†^ (0.68-2.16)	5.18 × 10^-1^	0.80^†^ (0.35-1.79)	5.79 × 10^-1^	1.93 (1.53-2.45)	4.99 × 10^-8^	79	0.01
**11p15**	*CHID1*	rs147862429	T	0.05	2.77^†^ (2.02-3.81)	2.90 × 10^-10^	1.34^†^ (0.71-2.53)	3.68 × 10^-1^	0.58^†^ (0.24-1.39)	2.23 × 10^-1^	2.09 (1.60-2.74)	7.87 × 10^-8^	85	0.001
**2q14**	*PCDP1*	rs139257032	T	0.02	2.78^†^ (1.57-4.92)	4.60 × 10^-4^	4.17^†^ (1.82-9.57)	7.41 × 10^-4^	10.79^†^ (1.27-91.39	2.91 × 10^-2^	3.35 (2.12-5.30)	2.53 × 10^-7^	0	0.4
**15q26**	*MCTP2*	rs12910412	G	0.46	1.29^†^ (1.14-1.46)	4.37 × 10^-5^	1.30^†^ (1.03-1.64)	2.49 × 10^-2^	1.33^†^ (0.98-1.78)	6.37 × 10^-2^	1.30 (1.17-1.44)	5.22 × 10^-7^	0	0.99
**11p15**	*CHID1*	rs139090846	T	0.01	4.65^†^ (2.77-7.80)	5.67 × 10^-9^	1.13^†^ (0.33-3.89)	8.41 × 10^-1^	0.75^†^ (0.19-2.91)	6.78 × 10^-1^	3.15 (2.01-4.94)	5.62 × 10^-7^	78	0.01
**11p15**	*AP2A2*	rs143705409	G	0.05	2.62^†^ (1.93-3.56)	7.19 × 10^-10^	1.32^†^ (0.73-2.40)	3.61 × 10^-1^	0.52^†^ (0.24-1.11)	8.95 × 10^-2^	1.92 (1.49-2.49)	6.16 × 10^-7^	88	0.0002
**11p15**	*AP2A2*	rs185786041	C	0.05	2.61^†^ (1.92-3.54)	8.44 × 10^-10^	1.31^†^ (0.73-2.35)	4.26 × 10^-1^	0.54^†^ (0.26-1.11)	9.50 × 10^-2^	1.89 (1.47-2.44)	9.20 × 10^-7^	88	0.0002
**11p15**	*AP2A2*	rs117455145	G	0.05	2.61^†^ (1.92-3.54)	8.50 × 10^-10^	1.31^†^ (0.73-2.34)	4.27 × 10^-1^	0.54^†^ (0.26-1.11)	9.28 × 10^-2^	1.89 (1.46-2.43)	9.68 × 10^-7^	88	0.0002

Definition of abbreviations: *CI* confidence interval, *FRQ* risk allele frequency, *NHW* non-Hispanic white, *OR* odds ratio, *SNP* single nucleotide polymorphism.

^*^Adjusted for age, sex, pack-years of cigarette smoking and genetic ancestry as summarized in the principal components.

^†^Imputed genotypes.

The novel locus on 11p15.5 encompasses a region where three genes are annotated: EF-hand calcium binding domain 4A (*EFCAB4A*), chitinase domain containing 1 (*CHID1*), and adaptor-related protein complex 2, alpha 2 subunit (*AP2A2*). The most significant SNP at this locus was rs34391416 (*EFCAB4A*), with a *P* value of 4.99 × 10^-8^. There was some evidence of heterogeneity (*P* = 0.01 for Cochran’s *Q*, *I*^*2*^ = 79). However, a meta-analysis using a modified random effects model showed more highly significant *P* values: *P* = 1.66 × 10^-8^ at rs34391416 (*EFCAB4A*), *P* = 7.56 × 10^-9^ at rs147862429 (*CHID1*), and *P* = 1.11 × 10^-8^ at rs143705409 (*AP2A2*).

### GWAS of CB COPD relative to COPD subjects without CB

**Figure 5:**
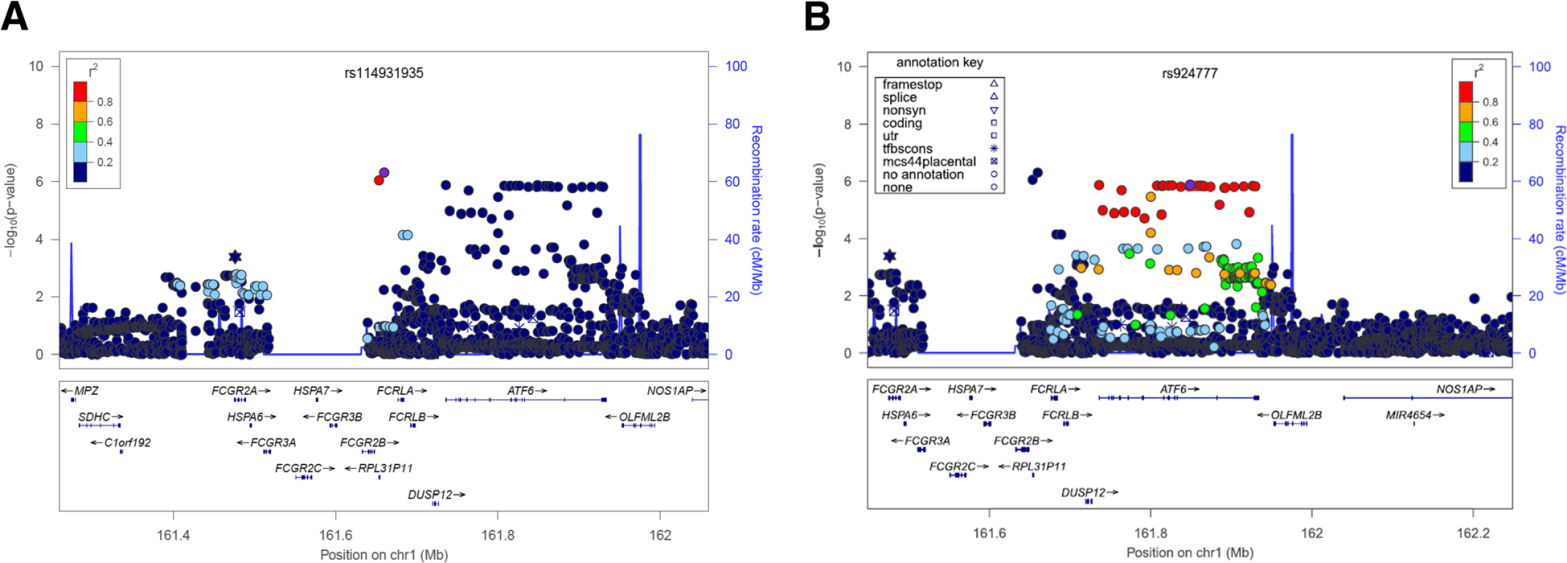
**Local association plots for the top two loci in the meta-analysis of COPD subjects with chronic bronchitis versus COPD subjects without chronic bronchitis in COPDGene non-Hispanic whites, GenKOLS, and ECLIPSE. A**. rs114931935 on 1q23. **B**. rs924777 on 1q23. The x-axis is chromosomal position, and the y-axis shows the –log10 P value. The most significant SNP at each gene is labeled in purple, with other SNPs colored by degree of linkage disequilibrium (r2). Plots were created using LocusZoom.

A GWAS of CB within COPD subjects from three studies included the same number of COPD CB cases and 3,777 COPD subjects without CB as a control group. Table 3 showed baseline characteristics of COPD subjects, and there is a corresponding Q-Q plot in Figure 2B (lambda = 1.01). We found a novel suggestive locus on 1q23.3, which did not reach genome-wide significant levels (rs114931935, *P* = 4.99 × 10^-7^, Table 4 and Figure 3B). This locus includes ribosomal protein L31 pseudogene 11 (*RPL31P11*) and activating transcription factor 6 (*ATF6*) (Figure 5).

**Table 3:** **Baseline characteristics of COPD subjects with chronic bronchitis (CB) and those without CB within each cohort**

	**COPDGene NHWs**		**GenKOLS**		**ECLIPSE**	
	**CB**	**No CB**	**CB**	**No CB**	**CB**	**No CB**
N	844	1968	311	552	507	1257
Age, years	62.8 (8.3)	65.5 (8.0)	65.3 (10.0)	65.7 (10.1)	62.3 (7.7)	64.2 (6.8)
Pack-years	59.2 (28.6)	55.1 (27.6)	34.0 (20.4)	30.9 (17.3)	51.6 (30.2)	49.8 (26.2)
Current smoker (%)	53.3	26.7	54.0	42.8	51.4	28.5
FEV_1_,% predicted	48.6 (17.4)	50.1 (18.2)	46.9 (16.8)	52.7 (17.5)	46.3 (15.3)	48.2 (15.7)
Sex (% male)	61.8	53.0	64.6	57.6	75.7	63.5

Data are presented as mean (SD) or percentage, as appropriate.

Definition of abbreviations: *CB* chronic bronchitis, *NHW* Non-Hispanic white.

**Table 4:** **Top results of the meta-analysis for chronic bronchitis (CB)** ***versus*** **no CB within COPD subjects of COPDGene non-Hispanic white, GenKOLS, and ECLIPSE studies** ^*^

**Locus**	**Nearest gene**	**SNP**	**Risk allele**	**FRQ**	**COPDGene NHWs**		**GenKOLS**		**ECLIPSE**		**Overall**			
					**OR (95% CI)**	***P***	**OR (95% CI)**	***P***	**OR (95% CI)**	***P***	**OR (95% CI)**	***P***	***I*** ^***2***^	***Q***
**1q23**	*RPL31P11*	rs114931935	A	0.04	1.98^†^ (1.42-2.78)	6.43 × 10^-5^	2.11^†^ (1.07-4.15)	3.10 × 10^-2^	1.64^†^ (1.07-2.51)	2.21 × 10^-2^	1.88 (1.47-2.40)	4.99 × 10^-7^	0	0.74
**1q23**	*RPL31P11*	rs114384494	T	0.04	2.01^†^ (1.43-2.84)	7.04 × 10^-5^	2.18^†^ (1.08-4.38)	2.92 × 10^-2^	1.61^†^ (1.03-2.51)	3.62 × 10^-2^	1.89 (1.47-2.44)	8.86 × 10^-7^	0	0.67
**1q23**	*ATF6*	rs924777	G	0.12	1.23^†^ (1.03-1.46)	2.20 × 10^-2^	1.21 (0.89-1.64)	2.19 × 10^-1^	1.69 (1.36-2.10)	2.30 × 10^-6^	1.36 (1.20-1.54)	1.33 × 10^-6^	65	0.06
**1q23**	*ATF6*	rs2298019	A	0.12	1.23^†^ (1.03-1.47)	1.98 × 10^-2^	1.25^†^ (0.93-1.70)	1.43 × 10^-1^	1.66^†^ (1.33-2.06)	6.41 × 10^-6^	1.36 (1.20-1.54)	1.36 × 10^-6^	56	0.10

Definition of abbreviations: *CI* confidence interval, *FRQ* risk allele frequency, *NHW* non-Hispanic white, *OR* odds ratio, *SNP* single nucleotide polymorphism.

^*^Adjusted for age, sex, pack-years of cigarette smoking and genetic ancestry as summarized in the principal components.

^†^Imputed genotypes.

Since the GWAS in COPD Gene NHWs for COPD with CB *versus* COPD without CB identified a genome-wide significant SNP, rs12692398 on 2p25.1, we performed a meta-analysis of two studies (COPDGene NHWs and GenKOLS), which also demonstrated the same SNP as a genome-wide significant SNP (Additional file 1: Table S1 and Additional file 1: Figure S1). It is located within cystin-1 (*CYS1*), encoding a cilia-associated protein. This SNP did not demonstrate evidence for association to CB within ECLIPSE COPD cases.

### Complementary analyses

To explore whether our results were similar when including an additional racial group, a supplemental meta-analysis of four cohorts by adding AAs of COPDGene was performed for CB COPD relative to smoking controls. Additional file 1: Table S2 shows the baseline characteristics of AAs of COPDGene. The meta-analysis including 1,844 cases and 5,269 controls revealed similar results to those of three cohorts, with the exception of SNPs in both *CHID* and *AP2A2,* which were excluded because of their rarity in AA subjects (minor allele frequency < 0.01). The novel top SNP, rs34391416 (*EFCAB4A*), was genome-wide significant (OR = 1.93, *P* = 2.66 × 10^-8^).

Since CB was present in some of our smoking controls, a GWAS of CB COPD *versus* smoking controls without CB (n = 3,101) was performed for each of our three cohorts and then meta-analyzed. These results were similar, although the novel top SNP on 11p15.5 was slightly reduced in statistical significance (rs34391416, OR = 1.98, *P* = 6.50 × 10^-8^). However, a meta-analysis of four cohorts including AAs of COPDGene (n = 4,628) showed genome-wide significance of the same SNP (OR = 1.98, *P* = 2.76 × 10^-8^). Baseline characteristics of smoking controls without CB were summarized in Additional file 1: Table S3.

Because COPD subjects with CB were more likely to be current smokers, complementary meta-analyses were performed with adjustment for current smoking status as well as age, gender, pack-years of cigarette smoking and genetic ancestry-based principal components. In meta-analyses using smokers with normal spirometry as a control group, *FAM13A* SNPs remained genome-wide significant. One of the previously reported COPD risk loci, 15q25, was nearly genome-wide significant (*P* = 6.58 × 10^-8^). However, the novel SNP on 11p15 (rs34391416) was not genome-wide significant (*P* = 5.25 × 10^-7^ in three Caucasian cohorts and *P* = 2.60 × 10^-7^ in four cohorts including AAs, Additional file 1: Table S4). On the other hand, a meta-analysis using COPD subjects without CB as a control group, with adjustment for current smoking status, provided lower (but not genome-wide significant) *P* values of top SNPs from the secondary analysis of COPD with CB vs. COPD without CB (Additional file 1: Table S5).

We assessed the top SNPs of CB COPD susceptibility relative to smokers with normal spirometry (the primary meta-analysis) in the results of the secondary meta-analysis of CB *vs*. no CB within COPD subjects. The novel SNPs on 11p15.5 were nominally significant (*P* < 0.01), whereas SNPs near *FAM13A* were not significant (*P* > 0.1) (Additional file 1: Table S6).

Clinical and radiological characteristics were compared according to genotypes of rs34391416 among all COPDGene NHW subjects (Additional file 1: Table S7). There were significant differences in parameters related to airway disease, including airway wall area% on inspiratory chest CT scans and gas trapping on expiratory CT. There were no differences in emphysema severity or distribution related to this SNP.

Since the meta-analysis of CB COPD relative to smoking controls showed *FAM13A* as the top gene, we performed additional analyses to ascertain whether SNPs near *FAM13A* had different levels of statistical significance between COPD with CB and COPD without CB. A meta-analysis of GWASs for COPD subjects without CB relative to smoking controls (Figure 1) also showed *FAM13A* as the top gene, which was followed by *HHIP* and *IREB2* (Additional file 1: Table S8 and Additional file 1: Figure S2). ORs and *P* values of previously known COPD risk alleles among our results from meta-analyses for CB COPD or COPD without CB are summarized in Table 5. Permutation testing revealed that differences of ORs between our two meta-analyses were statistically significant at four SNPs in *FAM13A*.

**Table 5:** **Meta-analysis results of COPD with chronic bronchitis (CB)** ***vs*** **. smoking controls and COPD without CB** ***vs*** **. smoking controls for COPD risk alleles previously demonstrated in genome-wide association studies of COPD** ***vs*** **. smoking controls**

**Locus**	**Gene**	**SNP**	**Risk allele**	**COPD with CB** ***vs*** **. smoking controls**			**COPD without CB** ***vs*** **. smoking controls**			**Permutation testing to assess difference of OR between two meta-analyses, 10,000 times**
				**FRQ**	**OR**	***P***	**FRQ**	**OR**	***P***	***P***
**4q22**	*FAM13A*	rs2869967^*^	C	0.41	1.38	5.61 × 10^-10^	0.41	1.29	9.73 × 10^-10^	1.10 × 10^-3^
**4q22**	*FAM13A*	rs4416442^†^	C	0.40	1.38	6.87 × 10^-10^	0.41	1.29	6.97 × 10^-10^	2.30 × 10^-3^
**4q22**	*FAM13A*	rs7671167^‡^	T	0.50	1.35	5.58 × 10^-9^	0.50	1.25	6.63 × 10^-8^	3.00 × 10^-4^
**4q22**	*FAM13A*	rs1964516^‡^	T	0.50	1.35	1.04 × 10^-8^	0.50	1.24	1.12 × 10^-7^	8.00 × 10^-4^
**4q31**	*HHIP-AS1*	rs13141641^†^	T	0.58	1.27	2.81 × 10^-6^	0.59	1.25	2.18 × 10^-8^	1.77 × 10^-1^
**4q31**	*HHIP-AS1*	rs13118928^‡^	A	0.58	1.24	2.72 × 10^-5^	0.59	1.25	3.15 × 10^-8^	4.54 × 10^-1^
**15q25**	*CHRNA3*	rs12914385^†^	T	0.42	1.27	2.83 × 10^-6^	0.42	1.22	7.86 × 10^-7^	5.75 × 10^-2^
**15q25**	*AGPHD1*	rs8034191^§^	C	0.37	1.27	4.29 × 10^-6^	0.37	1.22	1.34 × 10^-6^	5.13 × 10^-2^
**15q25**	*IREB2*	rs11858836^‡^	A	0.35	1.25	4.10 × 10^-5^	0.36	1.23	9.27 × 10^-7^	4.19 × 10^-1^
**19q13**	*RAB4B*	rs2604894^‡^	G	0.56	1.19	9.89 × 10^-4^	0.57	1.16	4.70 × 10^-4^	1.28 × 10^-1^
**14q32**	*RIN3*	rs754388^†^	C	0.81	1.22	3.74 × 10^-3^	0.82	1.29	2.37 × 10^-6^	5.84 × 10^-2^
**14q32**	*RIN3*	rs17184313^†^	C	0.82	1.20	6.85 × 10^-3^	0.83	1.28	4.36 × 10^-6^	1.92 × 10^-2^

Definition of abbreviations: *FRQ* risk allele frequency; *OR* odds ratio; *SNP* single nucleotide polymorphism.

^*^Cho MH, et al. Variants in FAM13A Are Associated With Chronic Obstructive Pulmonary Disease. *Nat Genet* 2010;42:200-202.

^†^Cho MH, et al. unpublished data.

^‡^Cho MH, et al. A Genome-Wide Association Study of COPD Identifies a Susceptibility Locus on Chromosome 19q13. *Hum Mol Genet* 2012;21:947-957.

^§^Pillai SG, et al. A Genome-Wide Association Study in Chronic Obstructive Pulmonary Disease (COPD): Identification of Two Major Susceptibility Loci. *PLoS Genet* 2009;5:e1000421.

## Discussion

Our GWAS meta-analysis of three studies of COPD subjects with CB relative to smoking controls not only reconfirmed previously known genome-wide significant SNPs in *FAM13A* related to lung function [32,33] and COPD [21-23], but also revealed a novel locus on 11p15.5, including *EFCAB4A*, *CHID1*, and *AP2A2.* Proteins encoded by one or more of these three genes could be involved in CB. Interestingly, this new region is located next to *MUC6* and *MUC2* (Figure 5) [34]. Thus, it is also possible that this genomic region influences regulation of mucin genes to alter susceptibility to CB.

*EFCAB4A* encodes a protein involved in store-operated Ca^2+^ entry. Intracellular Ca^2+^ was reported to regulate *MUC2* expression [34,35] and mucin secretion from airway goblet cells [36]. In addition, a study demonstrated increased intracellular Ca^2+^ levels in lymphocytes of COPD patients, which correlated positively with the spirometric grade of COPD [37]. Gene expression microarray analysis of human bronchial epithelial cells identified overexpression of *EFCAB4A* during mucociliary differentiation [38]. While quantitative RT-PCR revealed high expression of *EFCAB4A* in lung [39], a role for EFCAB4A in CB remains to be defined.

Even though SNPs near *CHID1* in the meta-analysis of three GWASs did not show genome-wide significance, rs147862429 was the most genome-wide significant in a GWAS of COPDGene NHWs, with a *P* value of 2.90 × 10^-10^. *CHID1* encodes a saccharide- and LPS-binding protein, also called stabilin-1 interacting chitinase-like protein (S1-CLP), with possible roles in pathogen sensing and endotoxin neutralization [40]. It is expressed in cells of monocytic, T lymphocyte, B lymphocyte, and epithelial origin, and it is up-regulated by the Th2 cytokine interleukin-4 and dexamethasone in macrophages [41]. Other human chitinase and chitinase-like proteins were previously suggested to play a role in the development of COPD [42]. Chitotriosidase (CHIT1) levels were elevated in the bronchoalveolar lavage fluid of smokers with COPD [43]. A chitinase-like protein, commonly known as YKL-40, was also increased in the lungs of COPD patients [44]. A recent study demonstrated genetic associations between chitinase gene variants and lung function level and rate of decline in COPD patients from the Lung Health Study [45]. Therefore, CHID1 may be involved in the pathogenesis of CB.

*AP2A2* encodes adaptor protein complex 2 subunit alpha-2, which has been shown to participate in the endocytosis of clathrin-coated vesicles in interacting with epsin-1 [46] and receptor endocytosis with SHC-transforming protein 1 [47]. One study demonstrated long-range interactions between the *MUC2* promoter and the adjacent *AP2A2* gene by using quantitative chromosome conformation capture (q3C) [48]. Although human respiratory tract mucus contains mainly MUC5AC and MUC5B along with smaller amounts of MUC2, the distribution of *MUC2* variable number tandem repeat (VNTR) alleles was reported to be different between asthmatics and non-asthmatics [49]. A follow-up study demonstrated relatively strong LD between SNPs in *MUC2* and *MUC5AC* [50]. Therefore, *AP2A2*, either alone or through interactions with *MUC2*, may have a potential role in CB pathogenesis.

In the primary meta-analysis of CB COPD relative to smoking controls, we found the strongest signal within *FAM13A* rather than the other known COPD susceptibility genes, and permutation testing confirmed that ORs of *FAM13A* SNPs were significantly higher than those for non-CB COPD. While COPD is a complex disease with marked phenotypic heterogeneity, most previous genetic studies have dealt with COPD subjects as one homogeneous group [20-22]. The current study suggests that previously identified COPD risk alleles might have different effects on the development of different COPD subtypes.

Although our secondary meta-analysis of CB COPD relative to COPD without CB within the COPD population failed to demonstrate genome-wide significant SNPs, the fourth most significant SNP, rs2298019, was previously identified as an expression quantitative trait locus (eQTL) for *ATF6* in lung tissue [51], with the risk allele associated with decreased expression. ATF6 plays a major role in transcriptional repression of endogenous cystic fibrosis transmembrane conductance regulator (*CFTR*) under endoplasmic reticulum stress [52] and is thought to be a potential therapeutic target for cystic fibrosis (CF) [53]. In addition to CF, suppressed CFTR function has been reported in cigarette smokers and COPD patients without CF [54,55]. Recently, roflumilast, approved to reduce COPD exacerbations in COPD patients with CB, has been reported to activate CFTR [56]. Since ATF6 is closely connected with CFTR, genetic variants of *ATF6* may play a role in the pathogenesis of CB.

We found that SNPs in another gene (*CYS1*) on 2p25.1 demonstrated suggestive associations for CB. CYS1 is enriched in the ciliary axoneme, and high expression in the kidney and weak expression in the lung were reported [57]. While the top SNP, rs12692398 of *CYS1,* reached the genome-wide significance threshold in both a GWAS of only COPDGene NHWs and a meta-analysis of COPDGene NHWs and GenKOLS, it lost significance in the meta-analysis of all three cohorts (*P* = 1.66 × 10^-4^). It is unclear why the association evidence for CB of this genomic region within ECLIPSE was negative. In the meta-analysis of three cohorts, the other two SNPs of *CYS1*, rs13000481 and rs4574084 showed *P* values of 1.74 × 10^-5^ and 2.61× 10^-5^, respectively, and LD between these two SNPs is high (0.94).

Our study has several limitations. First, we have not identified the functional genetic variants within our association regions. Nevertheless, we found significant differences in radiological parameters related to airway wall thickness according to genotypes of the novel top SNP, rs34391416, within COPDGene. These CT parameters have been frequently used as objective indicators of airway disease [6,58]. Interestingly, there were no differences in emphysema severity or distribution according to this SNP genotype. Further studies will be required to identify the functional genetic variants within this region and to determine which gene that they influence. Second, we have not performed any independent replication, although this analysis was a meta-analysis of three GWASs. However, a supplemental meta-analysis of four cohorts by adding COPDGene AAs also showed similar results as those of three cohorts. Third, CB was present in some of our smoking controls. However, an additional meta-analysis of three GWASs of CB COPD *versus* smoking controls without CB showed similar results.

## Conclusions

We have identified a novel locus on 11p15.5, which includes several biologically plausible candidates (*EFCAB4A*, *CHID1* and *AP2A2*) as potential CB susceptibility genes. We have also found significantly increased effect sizes of *FAM13A* SNPs in COPD subjects with CB compared to those without CB. Although our secondary GWAS of CB *versus* no CB within COPD subjects did not show genome-wide significant SNPs, a locus including *ATF6* should be explored for its related functional consequences. This study supports the concept that different genetic susceptibility contributes to phenotypic heterogeneity within COPD.

## Additional file

Additional file 1:
**Variable definitions.** Additional analysis methods. **Table S1.** Top results of the meta-analysis for COPD subjects with chronic bronchitis (CB) versus COPD subjects without CB in COPDGene non-Hispanic white and GenKOLS cohorts. **Table S2.** Baseline characteristics of COPD subjects with CB and smokers with normal spirometry as a control group in African Americans of COPDGene cohort. **Table S3.** Baseline characteristics of COPD subjects with CB and smoking controls without CB. **Table S4.** Top results of the two meta-analyses for COPD subjects with CB versus smokers with normal spirometry, including current smoking adjustment. **Table S5.** Top results of three Caucasian cohorts meta-analyses for COPD subjects with CB versus without CB, including current smoking adjustment. **Table S6.** Assessment of top results from COPD with CB versus smokers with normal spirometry within the meta-analysis for COPD subjects with CB versus without CB. **Table S7.** Clinical and radiological characteristics according to genotypes of rs34391416 among subjects with COPD and smoking controls among non-Hispanic whites of COPDGene. **Table S8.** Top results of three Caucasian cohorts meta-analyses for COPD subjects without CB versus smokers with normal spirometry. **Figure S1.** Local association plots for significant loci for the meta-analysis of COPD subjects with CB versus COPD subjects without CB in COPDGene non-Hispanic whites and GenKOLS. The x-axis is chromosomal position, and the y-axis shows the –log10 P value. The most significant SNP at each locus is labeled in purple, with other SNPs colored by degree of linkage disequilibrium (r2). Plots created using LocusZoom. **Figure S2.** (A) The quantile–quantile plot and (B) Manhattan plot of –log10 P values for the three-cohort meta-analysis including 1000 Genomes project imputed data for (A) COPD subjects without CB versus smoking controls after adjustment for age, sex, pack-years of cigarette smoking and genetic ancestry using principal components.

## Abbreviations

AA: African American
CB: Chronic bronchitis
ECLIPSE: Evaluation of COPD longitudinally to identify predictive surrogate endpoints
GOLD: Global initiative for chronic obstructive lung disease
GWAS: Genome-wide association study
NHW: non-Hispanic white
SNP: Single nucleotide polymorphism

## References

[1] Hallberg J, Dominicus A, Eriksson UK, Gerhardsson de Verdier M, Pedersen NL, Dahlback M, Nihlen U, Higenbottam T, Svartengren M (2008). Interaction between smoking and genetic factors in the development of chronic bronchitis. Am J Respir Crit Care Med.

[2] Burrows B, Fletcher CM, Heard BE, Jones NL, Wootliff JS (1966). The emphysematous and bronchial types of chronic airways obstruction. A clinicopathological study of patients in London and Chicago. Lancet.

[3] Ferre A, Fuhrman C, Zureik M, Chouaid C, Vergnenegre A, Huchon G, Delmas MC, Roche N (2012). Chronic bronchitis in the general population: influence of age, gender and socio-economic conditions. Respir Med.

[4] Patel BD, Coxson HO, Pillai SG, Agusti AG, Calverley PM, Donner CF, Make BJ, Muller NL, Rennard SI, Vestbo J, Wouters EF, Hiorns MP, Nakano Y, Camp PG, Nasute Fauerbach PV, Screaton NJ, Campbell EJ, Anderson WH, Paré PD, Levy RD, Lake SL, Silverman EK, Lomas DA, International COPD Genetics Network (2008). Airway wall thickening and emphysema show independent familial aggregation in chronic obstructive pulmonary disease. Am J Respir Crit Care Med.

[5] Kong X, Cho MH, Anderson W, Coxson HO, Muller N, Washko G, Hoffman EA, Bakke P, Gulsvik A, Lomas DA, Silverman EK, Pillai SG, ECLIPSE Study NETT Investigators (2011). Genome-wide association study identifies BICD1 as a susceptibility gene for emphysema. Am J Respir Crit Care Med.

[6] Kim V, Han MK, Vance GB, Make BJ, Newell JD, Hokanson JE, Hersh CP, Stinson D, Silverman EK, Criner GJ (2011). The chronic bronchitic phenotype of COPD: an analysis of the COPDGene Study. Chest.

[7] Kim V, Garfield JL, Grabianowski CL, Krahnke JS, Gaughan JP, Jacobs MR, Criner GJ (2011). The effect of chronic sputum production on respiratory symptoms in severe COPD. COPD.

[8] Burgel PR, Nesme-Meyer P, Chanez P, Caillaud D, Carre P, Perez T, Roche N (2009). Cough and sputum production are associated with frequent exacerbations and hospitalizations in COPD subjects. Chest.

[9] Silverman EK, Mosley JD, Palmer LJ, Barth M, Senter JM, Brown A, Drazen JM, Kwiatkowski DJ, Chapman HA, Campbell EJ, Province MA, Rao DC, Reilly JJ, Ginns LC, Speizer FE, Weiss ST (2002). Genome-wide linkage analysis of severe, early-onset chronic obstructive pulmonary disease: airflow obstruction and chronic bronchitis phenotypes. Hum Mol Genet.

[10] Zhu G, Agusti A, Gulsvik A, Bakke P, Coxson H, Lomas DA, Silverman EK, Pillai SG (2009). CTLA4 gene polymorphisms are associated with chronic bronchitis. Eur Respir J.

[11] Dijkstra AE, Smolonska J, van den Berge M, Wijmenga C, Zanen P, Luinge MA, Platteel M, Lammers JW, Dahlback M, Tosh K, Hiemstra PS, Sterk PJ, Spira A, Vestbo J, Nordestgaard BG, Benn M, Nielsen SF, Dahl M, Verschuren WM, Picavet HS, Smit HA, Owsijewitsch M, Kauczor HU, de Koning HJ, Nizankowska-Mogilnicka E, Mejza F, Nastalek P, van Diemen CC, Cho MH, Silverman EK (2014). Susceptibility to chronic mucus hypersecretion, a genome wide association study. PLoS One.

[12] Vestbo J, Anderson W, Coxson HO, Crim C, Dawber F, Edwards L, Hagan G, Knobil K, Lomas DA, MacNee W, Silverman EK, Tal-Singer R, ECLIPSE investigators (2008). Evaluation of COPD Longitudinally to Identify Predictive Surrogate End-points (ECLIPSE). Eur Respir J.

[13] Regan EA, Hokanson JE, Murphy JR, Make B, Lynch DA, Beaty TH, Curran-Everett D, Silverman EK, Crapo JD (2010). Genetic epidemiology of COPD (COPDGene) study design. COPD.

[14] Zhu G, Warren L, Aponte J, Gulsvik A, Bakke P, Anderson WH, Lomas DA, Silverman EK, Pillai SG (2007). The SERPINE2 gene is associated with chronic obstructive pulmonary disease in two large populations. Am J Respir Crit Care Med.

[15] Society AT (1962). Chronic bronchitis, asthma and pulmonary emphysema: a statement by the Committee on Diagnostic Standards for Nontuberculous Respiratory Diseases. Am Rev Respir Dis.

[16] Vestbo J, Hurd SS, Agusti AG, Jones PW, Vogelmeier C, Anzueto A, Barnes PJ, Fabbri LM, Martinez FJ, Nishimura M, Stockley RA, Sin DD, Rodriguez-Roisin R (2013). Global strategy for the diagnosis, management, and prevention of chronic obstructive pulmonary disease: GOLD executive summary. Am J Respir Crit Care Med.

[17] Li Y, Willer CJ, Ding J, Scheet P, Abecasis GR (2010). MaCH: using sequence and genotype data to estimate haplotypes and unobserved genotypes. Genet Epidemiol.

[18] Howie B, Fuchsberger C, Stephens M, Marchini J, Abecasis GR (2012). Fast and accurate genotype imputation in genome-wide association studies through pre-phasing. Nat Genet.

[19] Abecasis GR, Auton A, Brooks LD, DePristo MA, Durbin RM, Handsaker RE, Kang HM, Marth GT, McVean GA (2012). An integrated map of genetic variation from 1,092 human genomes. Nature.

[20] Pillai SG, Ge D, Zhu G, Kong X, Shianna KV, Need AC, Feng S, Hersh CP, Bakke P, Gulsvik A, Ruppert A, Lødrup Carlsen KC, Roses A, Anderson W, Rennard SI, Lomas DA, Silverman EK, Goldstein DB, ICGN Investigators (2009). A genome-wide association study in chronic obstructive pulmonary disease (COPD): identification of two major susceptibility loci. PLoS Genet.

[21] Cho MH, Boutaoui N, Klanderman BJ, Sylvia JS, Ziniti JP, Hersh CP, DeMeo DL, Hunninghake GM, Litonjua AA, Sparrow D, Lange C, Won S, Murphy JR, Beaty TH, Regan EA, Make BJ, Hokanson JE, Crapo JD, Kong X, Anderson WH, Tal-Singer R, Lomas DA, Bakke P, Gulsvik A, Pillai SG, Silverman EK (2010). Variants in FAM13A are associated with chronic obstructive pulmonary disease. Nat Genet.

[22] Cho MH, Castaldi PJ, Wan ES, Siedlinski M, Hersh CP, DeMeo DL, Himes BE, Sylvia JS, Klanderman BJ, Ziniti JP, Lange C, Litonjua AA, Sparrow D, Regan EA, Make BJ, Hokanson JE, Murray T, Hetmanski JB, Pillai SG, Kong X, Anderson WH, Tal-Singer R, Lomas DA, Coxson HO, Edwards LD, MacNee W, Vestbo J, Yates JC, Agusti A, Calverley PM (2012). A genome-wide association study of COPD identifies a susceptibility locus on chromosome 19q13. Hum Mol Genet.

[23] Cho MH, McDonald ML, Zhou X, Mattheisen M, Castaldi PJ, Hersh CP, Demeo DL, Sylvia JS, Ziniti J, Laird NM, Lange C, Litonjua AA, Sparrow D, Casaburi R, Barr RG, Regan EA, Make BJ, Hokanson JE, Lutz S, Dudenkov TM, Farzadegan H, Hetmanski JB, Tal-Singer R, Lomas DA, Bakke P, Gulsvik A, Crapo JD, Silverman EK, Beaty TH, NETT Genetics, ICGN, ECLIPSE and COPDGene Investigators (2014). Risk loci for chronic obstructive pulmonary disease: a genome-wide association study and meta-analysis. Lancet Respir Med.

[24] Purcell S, Neale B, Todd-Brown K, Thomas L, Ferreira MA, Bender D, Maller J, Sklar P, de Bakker PI, Daly MJ, Sham PC (2007). PLINK: a tool set for whole-genome association and population-based linkage analyses. Am J Hum Genet.

[25] de Bakker PI, Ferreira MA, Jia X, Neale BM, Raychaudhuri S, Voight BF (2008). Practical aspects of imputation-driven meta-analysis of genome-wide association studies. Hum Mol Genet.

[26] Willer CJ, Li Y, Abecasis GR (2010). METAL: fast and efficient meta-analysis of genomewide association scans. Bioinformatics.

[27] Higgins JP, Thompson SG, Deeks JJ, Altman DG (2003). Measuring inconsistency in meta-analyses. BMJ.

[28] Han B, Eskin E (2011). Random-effects model aimed at discovering associations in meta-analysis of genome-wide association studies. Am J Hum Genet.

[29] Devlin B, Roeder K (1999). Genomic control for association studies. Biometrics.

[30] Aulchenko YS, Ripke S, Isaacs A, van Duijn CM (2007). GenABEL: an R library for genome-wide association analysis. Bioinformatics.

[31] Pruim RJ, Welch RP, Sanna S, Teslovich TM, Chines PS, Gliedt TP, Boehnke M, Abecasis GR, Willer CJ (2010). LocusZoom: regional visualization of genome-wide association scan results. Bioinformatics.

[32] Hancock DB, Artigas MS, Gharib SA, Henry A, Manichaikul A, Ramasamy A, Loth DW, Imboden M, Koch B, McArdle WL, Smith AV, Smolonska J, Sood A, Tang W, Wilk JB, Zhai G, Zhao JH, Aschard H, Burkart KM, Curjuric I, Eijgelsheim M, Elliott P, Gu X, Harris TB, Janson C, Homuth G, Hysi PG, Liu JZ, Loehr LR, Lohman K (2012). Genome-wide joint meta-analysis of SNP and SNP-by-smoking interaction identifies novel loci for pulmonary function. PLoS Genet.

[33] Hancock DB, Eijgelsheim M, Wilk JB, Gharib SA, Loehr LR, Marciante KD, Franceschini N, van Durme YM, Chen TH, Barr RG, Schabath MB, Couper DJ, Brusselle GG, Psaty BM, van Duijn CM, Rotter JI, Uitterlinden AG, Hofman A, Punjabi NM, Rivadeneira F, Morrison AC, Enright PL, North KE, Heckbert SR, Lumley T, Stricker BH, O'Connor GT, London SJ (2010). Meta-analyses of genome-wide association studies identify multiple loci associated with pulmonary function. Nat Genet.

[34] Rose MC, Voynow JA (2006). Respiratory tract mucin genes and mucin glycoproteins in health and disease. Physiol Rev.

[35] Thai P, Loukoianov A, Wachi S, Wu R (2008). Regulation of airway mucin gene expression. Annu Rev Physiol.

[36] Rossi AH, Salmon WC, Chua M, Davis CW (2007). Calcium signaling in human airway goblet cells following purinergic activation. Am J Physiol Lung Cell Mol Physiol.

[37] Manral S, Bhatia S, Sinha R, Kumar A, Rohil V, Arya A, Dhawan A, Arya P, Joshi R, Sreedhara SC, Gangopadhyay S, Bansal SK, Chatterjee S, Chaudhury NK, Vijayan VK, Saso L, Parmar VS, DePass AL, Prasad AK, Raj HG (2011). Normalization of deranged signal transduction in lymphocytes of COPD patients by the novel calcium channel blocker H-DHPM. Biochimie.

[38] Ross AJ, Dailey LA, Brighton LE, Devlin RB (2007). Transcriptional profiling of mucociliary differentiation in human airway epithelial cells. Am J Respir Cell Mol Biol.

[39] Yanai I, Benjamin H, Shmoish M, Chalifa-Caspi V, Shklar M, Ophir R, Bar-Even A, Horn-Saban S, Safran M, Domany E, Lancet D, Shmueli O (2005). Genome-wide midrange transcription profiles reveal expression level relationships in human tissue specification. Bioinformatics.

[40] Meng G, Zhao Y, Bai X, Liu Y, Green TJ, Luo M, Zheng X (2010). Structure of human stabilin-1 interacting chitinase-like protein (SI-CLP) reveals a saccharide-binding cleft with lower sugar-binding selectivity. J Biol Chem.

[41] Kzhyshkowska J, Mamidi S, Gratchev A, Kremmer E, Schmuttermaier C, Krusell L, Haus G, Utikal J, Schledzewski K, Scholtze J, Goerdt S (2006). Novel stabilin-1 interacting chitinase-like protein (SI-CLP) is up-regulated in alternatively activated macrophages and secreted via lysosomal pathway. Blood.

[42] Lee CG, Da Silva CA, Lee JY, Hartl D, Elias JA (2008). Chitin regulation of immune responses: an old molecule with new roles. Curr Opin Immunol.

[43] Letuve S, Kozhich A, Humbles A, Brewah Y, Dombret MC, Grandsaigne M, Adle H, Kolbeck R, Aubier M, Coyle AJ, Pretolani M (2010). Lung chitinolytic activity and chitotriosidase are elevated in chronic obstructive pulmonary disease and contribute to lung inflammation. Am J Pathol.

[44] Letuve S, Kozhich A, Arouche N, Grandsaigne M, Reed J, Dombret MC, Kiener PA, Aubier M, Coyle AJ, Pretolani M (2008). YKL-40 is elevated in patients with chronic obstructive pulmonary disease and activates alveolar macrophages. J Immunol.

[45] Aminuddin F, Akhabir L, Stefanowicz D, Pare PD, Connett JE, Anthonisen NR, Fahy JV, Seibold MA, Burchard EG, Eng C, Gulsvik A, Bakke P, Cho MH, Litonjua A, Lomas DA, Anderson WH, Beaty TH, Crapo JD, Silverman EK, Sandford AJ (2012). Genetic association between human chitinases and lung function in COPD. Hum Genet.

[46] Chen H, Fre S, Slepnev VI, Capua MR, Takei K, Butler MH, Di Fiore PP, De CP (1998). Epsin is an EH-domain-binding protein implicated in clathrin-mediated endocytosis. Nature.

[47] Okabayashi Y, Sugimoto Y, Totty NF, Hsuan J, Kido Y, Sakaguchi K, Gout I, Waterfield MD, Kasuga M (1996). Interaction of Shc with adaptor protein adaptins. J Biol Chem.

[48] Gosalia N, Leir SH, Harris A (2013). Coordinate regulation of the gel-forming mucin genes at chromosome 11p15.5. J Biol Chem.

[49] Vinall LE, Fowler JC, Jones AL, Kirkbride HJ, de BC, Laine A, Porchet N, Gum JR, Kim YS, Moss FM, Mitchell DM, Swallow DM (2000). Polymorphism of human mucin genes in chest disease: possible significance of MUC2. Am J Respir Cell Mol Biol.

[50] Rousseau K, Byrne C, Griesinger G, Leung A, Chung A, Hill AS, Swallow DM (2007). Allelic association and recombination hotspots in the mucin gene (MUC) complex on chromosome 11p15.5. Ann Hum Genet.

[51] Hao K, Bosse Y, Nickle DC, Pare PD, Postma DS, Laviolette M, Sandford A, Hackett TL, Daley D, Hogg JC, Elliott WM, Couture C, Lamontagne M, Brandsma CA, van den Berge M, Koppelman G, Reicin AS, Nicholson DW, Malkov V, Derry JM, Suver C, Tsou JA, Kulkarni A, Zhang C, Vessey R, Opiteck GJ, Curtis SP, Timens W, Sin DD (2012). Lung eQTLs to help reveal the molecular underpinnings of asthma. PLoS Genet.

[52] Bartoszewski R, Rab A, Twitty G, Stevenson L, Fortenberry J, Piotrowski A, Dumanski JP, Bebok Z (2008). The mechanism of cystic fibrosis transmembrane conductance regulator transcriptional repression during the unfolded protein response. J Biol Chem.

[53] Kerbiriou M, Le Drevo MA, Ferec C, Trouve P (2007). Coupling cystic fibrosis to endoplasmic reticulum stress: differential role of Grp78 and ATF6. Biochim Biophys Acta.

[54] Cantin AM, Hanrahan JW, Bilodeau G, Ellis L, Dupuis A, Liao J, Zielenski J, Durie P (2006). Cystic fibrosis transmembrane conductance regulator function is suppressed in cigarette smokers. Am J Respir Crit Care Med.

[55] Dransfield MT, Wilhelm AM, Flanagan B, Courville C, Tidwell SL, Raju SV, Gaggar A, Steele C, Tang LP, Liu B, Rowe SM (2013). Acquired cystic fibrosis transmembrane conductance regulator dysfunction in the lower airways in COPD. Chest.

[56] Lambert JA, Raju SV, Tang LP, McNicholas CM, Li Y, Courville CA, Farris RF, Coricor GE, Smoot LH, Mazur MM, Dransfield MT, Bolger GB, Rowe SM (2014). Cystic fibrosis transmembrane conductance regulator activation by roflumilast contributes to therapeutic benefit in chronic bronchitis. Am J Respir Cell Mol Biol.

[57] Fliegauf M, Frohlich C, Horvath J, Olbrich H, Hildebrandt F, Omran H (2003). Identification of the human CYS1 gene and candidate gene analysis in Boichis disease. Pediatr Nephrol.

[58] Orlandi I, Moroni C, Camiciottoli G, Bartolucci M, Pistolesi M, Villari N, Mascalchi M (2005). Chronic obstructive pulmonary disease: thin-section CT measurement of airway wall thickness and lung attenuation. Radiology.

