# Supplementary material for: Genetic susceptibility for chronic bronchitis in chronic obstructive pulmonary disease
Source: Respir Res. 2014 Sep 21;15(1):113. doi: 10.1186/s12931-014-0113-2 (PMC4190389; doi:10.1186/s12931-014-0113-2)

Supplemental Materials

Genetic Susceptibility for Chronic Bronchitis in Chronic Obstructive Pulmonary Disease

Jin Hwa Lee, Michael H. Cho, Craig P. Hersh, Merry-Lynn N. McDonald, James D. Crapo, Per S. Bakke, Amund Gulsvik, Alejandro P. Comellas, Christine H. Wendt, David A. Lomas, Victor Kim, Edwin K. Silverman, on behalf of the COPDGene and ECLIPSE Investigators

e-Appendix 1. Variable definitions

In the COPDGene study, quantitative CT measurements were performed using Slicer (Version 2, www.slicer.org). Percent emphysema was calculated as the percentage of voxels within the lung with an attenuation < −950 Hounsfield units (HU) at full inspiration [% low attenuation area (LAA)-950_{insp}]. Airway analysis was performed using the Pulmonary Workstation Plus (VIDA Diagnostics, Inc., Coralville, IA). In each segmental bronchus, the wall area (WA) and lumen area (LA) were measured. The segmental wall area % was defined as 100 × WA/ (WA + LA). The square roots of the wall area for hypothetical airways with an internal perimeter of 10 mm (Pi10) and 15 mm (Pi15) were used as measures of airway disease. Total lung capacity (TLC_{CT}) in liters was calculated from volumetric CT measurements at full inspiration. Gas trapping was quantified as the percentage of lung volume on expiratory CT with an attenuation < −856 HU (%LAA-856_{exp}).

e-Appendix 2. Additional analysis methods

We used permutation testing to assess differences in odds ratios (ORs) of previous known genome-wide significant SNPs between two meta-analyses for COPD subjects with CB and those without CB. For each cohort, we randomly reassigned the phenotypes (COPD with CB or without CB) of each individual to another individual in the dataset. Each random reassignment of the data represents one possible sampling of individuals under the null hypothesis, and this process is repeated a predefined number of times N to generate an empirical distribution with resolution N. Logistic regression was performed and the results were combined using meta-analysis. We repeated this procedure 10,000 times to obtain the null distribution of differences of effect sizes. Our baseline difference of effect size for each SNP was compared the permutation results, which was described by the null distribution to obtain a *P* value.

e-Table 1. Top results of the meta-analysis for COPD subjects with chronic bronchitis *versus* COPD subjects without chronic bronchitis in COPDGene non-Hispanic white and GenKOLS cohorts*

Locus	Nearest gene	SNP	Risk Allele	FRQ	COPDGene NHWs		GenKOLS		Overall			
					OR (95% CI)	*P*	OR (95% CI)	*P*	OR (95% CI)	*P*	I^2	*Q*
2p25	*CYS1*	rs12692398	A	0.20	1.53† (1.32-1.77)	1.57×10^{-8}	1.17† (0.90-1.54)	2.42×10^{-1}	1.44 (1.26-1.64)	3.33×10^{-8}	65	0.09
2p25	*CYS1*	rs34900318	A	0.16	1.55† (1.32-1.81)	9.44×10^{-8}	1.12† (0.85-1.48)	4.30×10^{-1}	1.43 (1.24-1.64)	5.01×10^{-7}	74	0.05
8p23	*CSMD1*	rs142700918	G	0.93	1.70† (1.31-2.20)	5.60×10^{-5}	2.14† (1.27-3.60)	4.06×10^{-3}	1.78 (1.41-2.25)	1.02×10^{-6}	0	0.44
2p25	*CYS1*	rs4536628	C	0.27	1.44† (1.26-1.65)	1.37×10^{-7}	1.07† (0.84-1.35)	5.96×10^{-1}	1.34 (1.19-1.51)	1.35×10^{-6}	79	0.03
2p25	*CYS1*	rs4257371	A	0.22	1.45† (1.26-1.68)	2.34×10^{-7}	1.09† (0.85-1.39)	5.03×10^{-1}	1.35 (1.20-1.53)	1.47×10^{-6}	75	0.05

Definition of abbreviations: CI = confidence interval; FRQ = risk allele frequency; NHW = non-Hispanic white; OR = odds ratio; SNP = single nucleotide polymorphism.

*Adjusted for age, sex, pack-years of cigarette smoking and genetic ancestry as summarized in the principal components.

†Imputed genotypes

e-Table 2. Baseline characteristics of COPD subjects with chronic bronchitis and smokers with normal spirometry as a control group in African Americans of COPDGene cohort.

	COPDGene African Americans	
	COPD with chronic bronchitis	Controls
n	182	1,749
Age, years	57.8 (7.4)	52.8 (6.0)
Pack-years	44.2 (24.0)	36.4 (20.1)
Current Smoker (%)	73.6	87.4
FEV_1, % predicted	52.6 (16.6)	98.4 (12.2)
Sex (% male)	59.3	58.1

Data are presented as mean (SD) or percentage, as appropriate.

e-Table 3. Baseline characteristics of COPD subjects with chronic bronchitis and smoking controls without chronic bronchitis

	COPDGene NHWs		GenKOLS		ECLIPSE		COPDGene AAs	
	COPD with CB	Controls without CB	COPD with CB	Controls without CB	COPD with CB	Controls without CB	COPD with CB	Controls without CB
n	844	2,217	311	730	507	154	182	1,527
Age, years	62.8 (8.3)	59.9 (8.7)	65.3 (10.0)	55.5 (9.6)	62.3 (7.7)	57.4 (9.4)	57.8 (7.4)	52.8 (6.1)
Pack-years	59.2 (28.6)	37.3 (20.1)	33.9 (20.2)	19.1 (13.0)	51.6 (30.2)	32.4 (26.3)	44.2 (24.0)	35.8 (19.5)
Current smoker (%)	53.3	34.9	54.0	40.4	51.4	36.6	73.6	87.0
FEV_1, % predicted	48.6 (17.4)	97.0 (11.0)	46.9 (16.8)	95.1 (9.3)	46.3 (15.3)	107.7 (13.6)	52.6 (16.6)	98.6 (12.2)
Sex (% male)	61.8	48.3	64.6	49.3	75.7	61.7	59.3	58.9

Data are presented as mean (SD) or percentage, as appropriate.
Definition of abbreviations: AA = African American; CB = chronic bronchitis; NHW = non-Hispanic white.

e-Table 4. Top results of the two meta-analyses for COPD subjects with chronic bronchitis *versus* smokers with normal spirometry, including current smoking adjustment*

Locus	Nearest gene	SNP	Risk Allele	FRQ		COPDGene NHWs, GenKOLS, and ECLIPSE cohorts				COPDGene NHWs and AAs, GenKOLS, and ECLIPSE cohorts			
				EUR	AA	OR (95% CI)	*P*	I^2	*Q*	OR (95% CI)	*P*	I^2	*Q*
4q22	*FAM13A*	rs1812329		0.40	058	1.40 (1.26-1.56)	1.91×10^{-10}	0	0.47	1.35 (1.23-1.49)	4.04×10^{-10}	28	0.25
4q22	*FAM13A*	rs4416442		0.40	0.54	1.40 (1.26-1.56)	2.32×10^{-10}	0	0.47	1.36 (1.24-1.50)	1.57×10^{-10}	4	0.37
15q25	*AGPHD1*	rs9788721		0.36	0.37	1.34 (1.21-1.49)	6.58×10^{-8}	29	0.25	1.32 (1.20-1.45)	2.76×10^{-8}	12	0.33
15q25	*CHRNA3*	rs12914385		0.42	0.19	1.31 (1.18-1.45)	2.70×10^{-7}	36	0.21	1.32 (1.20-1.45)	2.17×10^{-8}	6	0.36
11p15	*EFCAB4A*	rs34391416		0.05	0.01	1.85 (1.45-2.34)	5.25×10^{-7}	81	0.006	1.85 (1.46-2.34)	2.60×10^{-7}	71	0.02

Definition of abbreviations: AA = African American; CI = confidence interval; EUR = European white; FRQ = risk allele frequency; NHW = non-Hispanic white; OR = odds ratio; SNP = single nucleotide polymorphism.

*Adjusted for age, sex, current smoking, pack-years of cigarette smoking and genetic ancestry as summarized in the principal components.

e-Table 5. Top results of three Caucasian cohorts meta-analyses for COPD subjects with chronic bronchitis (CB) *versus* without CB, including current smoking adjustment*

Locus	Nearest gene	SNP	Risk Allele	FRQ	COPDGene NHWs		GenKOLS		ECLIPSE		Overall			
					OR (95% CI)	*P*	OR (95% CI)	*P*	OR (95% CI)	*P*	OR (95% CI)	*P*	I^2	*Q*
1q23	*RPL31P11*	rs114931935	A	0.04	2.15† (1.53-3.03)	1.23×10^{-5}	2.05† (1.04-4.04)	3.89×10^{-2}	1.57† (1.01-2.44)	4.59×10^{-2}	1.93 (1.50-2.48)	3.22×10^{-7}	0	0.53
1p22	*LOC100505768*	rs17423694	G	0.91	1.52† (1.22-1.90)	2.39×10^{-4}	1.16† (0.80-1.68)	4.27×10^{-1}	1.93† (1.39-2.70)	1.04×10^{-4}	1.53 (1.29-1.80)	5.35×10^{-7}	51	0.13
1q23	*RPL31P11*	rs114384494	T	0.04	2.18† (1.53-3.10)	1.50×10^{-5}	2.12† (1.05-4.28)	3.59×10^{-2}	1.54† (0.97-2.45)	6.54×10^{-2}	1.95 (1.50-2.53)	5.37×10^{-7}	0	0.49
1q23	*ATF6*	rs2499856	A	0.12	1.30† (1.08-1.55)	4.59×10^{-3}	1.24† (0.91-1.68)	1.67×10^{-1}	1.61† (1.29-2.02)	3.12×10^{-5}	1.38 (1.21-1.57)	7.34×10^{-7}	28	0.25
1q23	*ATF6*	rs2298019	A	0.12	1.28† (1.07-1.54)	6.93×10^{-3}	1.26† (0.93-1.70)	1.41×10^{-1}	1.62† (1.29-2.03)	2.92×10^{-5}	1.38 (1.21-1.57)	9.69×10^{-7}	31	0.23

Definition of abbreviations: CI = confidence interval; FRQ = risk allele frequency; NHW = non-Hispanic white; OR = odds ratio; SNP = single nucleotide polymorphism.
*Adjusted for age, sex, current smoking, pack-years of cigarette smoking and genetic ancestry as summarized in the principal components.

e-Table 6. Assessment of top results from COPD with CB *versus* smokers with normal spirometry within the meta-analysis for COPD subjects with CB *versus* without CB[*]

Locus	Nearest gene	SNP	Risk Allele	FRQ	COPDGene		GenKOLS		ECLIPSE		Overall			
					OR (95% CI)	*P*	OR (95% CI)	*P*	OR (95% CI)	*P*	OR (95% CI)	*P*	I^2	*Q*
4q22	*FAM13A*	rs2869967	C	0.41	1.02 (0.91-1.15)	7.17×10^{-1}	1.27 (1.03-1.55)	2.26×10^{-2}	0.95 (0.81-1.10)	4.75×10^{-1}	1.04 (0.95-1.13)	4.14×10^{-1}	61	0.08
4q22	*FAM13A*	rs2045517	T	0.44	1.02[†] (0.91-1.15)	7.15×10^{-1}	1.27[†] (1.03-1.55)	2.31×10^{-2}	0.95[†] (0.81-1.10)	4.74×10^{-1}	1.04 (0.95-1.13)	4.16×10^{-1}	61	0.08
4q22	*FAM13A*	rs7671167	T	0.53	1.08 (0.96-1.21)	2.20×10^{-1}	1.11 (0.91-1.35)	3.12×10^{-1}	0.96 (0.82-1.11)	5.55×10^{-1}	1.04 (0.96-1.14)	3.28×10^{-1}	0	0.38
4q22	*FAM13A*	rs2904259	C	0.53	1.08[†] (0.96-1.21)	2.10×10^{-1}	1.11[†] (0.91-1.35)	3.12×10^{-1}	0.95[†] (0.82-1.11)	5.55×10^{-1}	1.04 (0.96-1.14)	3.17×10^{-1}	0	0.38
11p15	*EFCAB4A*	rs34391416	A	0.04	1.53 (1.18-1.97)	1.26×10^{-3}	1.27[†] (0.76-2.11)	3.63×10^{-1}	1.37[†] (0.87-2.16)	1.79×10^{-1}	1.45 (1.18-1.78)	3.92×10^{-4}	0	0.79
11p15	*CHID1*	rs147862429	T	0.05	1.71[†] (1.27-2.30)	3.75×10^{-4}	1.40[†] (0.79-2.49)	2.48×10^{-1}	1.24[†] (0.75-2.06)	3.98×10^{-1}	1.55 (1.22-1.95)	2.47×10^{-4}	0	0.53
2q14	*PCDP1*	rs139257032	T	0.02	2.05[†] (1.23-3.43)	6.22×10^{-3}	2.03[†] (1.01-4.10)	4.72×10^{-2}	1.74[†] (0.83-3.64)	1.43×10^{-1}	1.97 (1.37-2.82)	2.47×10^{-4}	0	0.93
15q26	*MCTP2*	rs12910412	G	0.47	1.11[†] (0.98-1.25)	8.98×10^{-2}	1.26[†] (1.02-1.57)	3.39×10^{-2}	1.03[†] (0.88-1.22)	6.94×10^{-1}	1.11 (1.02-1.21)	1.98×10^{-2}	5	0.35
11p15	*CHID1*	rs139090846	T	0.02	1.80[†] (1.14-2.85)	1.18×10^{-4}	1.34[†] (0.47-3.81)	5.80×10^{-1}	2.56[†] (1.10-5.93)	2.85×10^{-2}	1.86 (1.28-2.71)	1.19×10^{-3}	0	0.62
11p15	*AP2A2*	rs143705409	G	0.05	1.71[†] (1.28-2.28)	2.71×10^{-4}	1.29[†] (0.47-2.25)	3.67×10^{-1}	1.11[†] (0.71-1.75)	6.37×10^{-1}	1.47 (1.18-1.84)	6.58×10^{-4}	26	0.26
11p15	*AP2A2*	rs185786041	C	0.05	1.70[†] (1.28-2.27)	2.92×10^{-4}	1.26[†] (0.73-2.16)	4.01×10^{-1}	1.12[†] (0.73-1.72)	6.14×10^{-1}	1.45 (1.17-1.81)	8.06×10^{-4}	30	0.24
11p15	*AP2A2*	rs117455145	G	0.05	1.70[†] (1.28-2.27)	2.93×10^{-4}	1.26[†] (0.73-2.16)	4.02×10^{-1}	1.11[†] (0.73-1.71)	6.22×10^{-1}	1.45 (1.17-1.81)	8.35×10^{-4}	31	0.24

Definition of abbreviations: CI = confidence interval; FRQ = risk allele frequency; OR = odds ratio; SNP = single nucleotide polymorphism.
[*]Adjusted for age, sex, pack-years of cigarette smoking and genetic ancestry as summarized in the principal components.
[†]Imputed genotypes

e-Table 7. Clinical and radiological characteristics according to genotypes of rs34391416 among subjects with COPD and smoking controls among non-Hispanic whites of COPDGene (N=5,339)

Genotype	GG	AG	AA	P^*
n	4,908	423	8	
Age, years	62.2 (8.85)	62.6 (8.7)	58.2 (6.5)	NS
Female gender, %	47.3	48.2	37.5	NS
Pack-years of smoking	47.4 (26.2)	49.5 (27.5)	51.0 (31.0)	NS
Current smoker, %	36.9	39.0	50.0	NS
Body mass index, kg/m^2	28.5 (5.9)	28.5 (6.4)	29.1 (7.2)	NS
6-min walked distance, feet	1389 (403)	1353 (410)	1518 (265)	NS
Exacerbation frequency per yr	0.46 (1.01)	0.59 (1.14)	1.50 (2.27)	0.0014
Severe exacerbation, %	11.7	13.2	25.0	NS
Chronic bronchitis, %	21.0	30.0	37.5	5.08×10^{-5}
Total lung capacity $(TLC)_{CT}$, L	5.94 (1.36)	6.02 (1.30)	6.53 (1.35)	NS
TLC % predicted	99.1 (15.8)	100.7 (16.0)	105.2 (10.1)	0.038
% emphysema	8.3 (11.0)	8.8 (11.3)	7.7 (12.8)	NS
% emphysema upper third	9.4 (14.3)	10.5 (15.1)	9.1 (15.9)	NS
% emphysema lower third	7.2 (9.4)	7.3 (9.1)	6.4 (8.4)	NS
% emphysema upper 3rd/lower 3rd ratio	1.8 (8.2)	1.7 (3.0)	1.0 (0.7)	NS
% gas trapping	26.3 (21.6)	29.1 (21.9)	23.3 (20.9)	$0.044^†$
Segmental wall area %	61.3 (3.2)	61.8 (3.2)	62.1 (4.3)	0.003
Subsegmental wall area %	64.3 (2.6)	64.8 (2.7)	67.0 (2.9)	$0.027^†$
Pi10	3.67 (0.13)	3.68 (0.13)	3.70 (0.19)	$NS^†$
Pi15	5.14 (0.19)	5.16 (0.19)	5.23 (0.36)	$0.048^†$
FEV_1 % predicted	72.4 (28.0)	66.8 (26.8)	80.3 (36.8)	0.0004
FVC % predicted	85.5 (17.5)	83.2 (16.9)	92.4 (24.0)	0.033
FEV_1/FVC	0.63 (0.18)	0.60 (0.18)	0.63 (0.17)	0.001
$FEF_{25-75\%}$, L/min	1.61 (1.30)	1.3 (1.2)	1.87 (1.59)	0.0002
Positive bronchodilator response, %	23.0	29.6	25.0	0.009

Data are presented as mean (standard deviation) or percent.
*ANOVA except for †Kruskal-Wallis test
Bronchodilator responsiveness was considered positive if the change in FEV1 or FVC was ≥200mL and ≥ 12% predicted following administration of short-acting inhaled beta-agonist.
For 6-min walked distance, n = 5,240
For TLC_{CT} and % emphysema, n = 5,043.
For TLC % predicted, n=5,046.
For emphysema upper third, lower third, upper 3rd/lower 3rd ratio, n=4,950.
For % gas trapping, n = 4,649. For % gas trapping except for data from the University of Iowa, n=3,759.
For segmental wall area %, n = 5,044.
For subsegmental wall area %, n = 2,049.
For Pi10 and Pi15, n = 4,923.
For bronchodilator response, n = 5,302.

e-Table 8. Top results of the meta-analysis for COPD cases without chronic bronchitis *versus* smokers with normal spirometry in COPDGene non-Hispanic white, GenKOLS, and ECLIPSE studies[*]

Locus	Nearest gene	SNP	Risk Allele	Freq	COPDGene		GenKOLS		ECLIPSE		Overall			
					OR (95% CI)	*P*	OR (95% CI)	*P*	OR (95% CI)	*P*	OR (95% CI)	*P*	I^2	*Q*
4q22	*FAM13A*	rs2869966	T	0.41	1.27 (1.16-1.40)	8.72×10^{-7}	1.29[†] (1.07-1.55)	7.64×10^{-3}	1.44[†] (1.11-1.86)	6.31×10^{-3}	1.29 (1.19-1.40)	6.77×10^{-10}	0	0.69
4q22	*FAM13A*	rs7682317	T	0.40	1.27[†] (1.16-1.40)	8.28×10^{-7}	1.29[†] (1.07-1.55)	7.87×10^{-3}	1.43[†] (1.11-1.86)	6.55×10^{-3}	1.29 (1.19-1.40)	6.82×10^{-10}	0	0.70
4q31	*HHIP-AS1*	rs138641402	A	0.63	1.27[†] (1.15-1.41)	5.38×10^{-6}	1.35[†] (1.10-1.65)	3.54×10^{-3}	1.40[†] (1.07-1.84)	1.56×10^{-2}	1.30 (1.19-1.42)	4.40×10^{-9}	0	0.75
4q31	*HHIP-AS1*	rs1980057	C	0.59	1.23 (1.12-1.35)	1.45×10^{-5}	1.29[†] (1.08-1.55)	5.49×10^{-3}	1.31[†] (1.03-1.68)	3.04×10^{-2}	1.25 (1.16-1.35)	2.84×10^{-8}	0	0.83
15q25	*IREB2*	rs8042238	T	0.64	1.26[†] (1.15-1.39)	3.07×10^{-6}	1.23[†] (1.01-1.49)	3.47×10^{-2}	1.28[†] (0.99-1.65)	5.96×10^{-2}	1.26 (1.16-1.37)	5.09×10^{-8}	0	0.96
15q25	*IREB2*	rs13180	T	0.64	1.25 (1.14-1.38)	5.92×10^{-6}	1.24 (1.03-1.50)	2.66×10^{-2}	1.27 (0.98-1.63)	6.78×10^{-2}	1.25 (1.15-1.36)	8.25×10^{-8}	0	0.99

Definition of abbreviations: CI: confidence interval; Freq = risk allele frequency; OR = odds ratio; SNP = single nucleotide polymorphism.
[*]Adjusted for age, sex, pack-years of cigarette smoking and genetic ancestry as summarized in the principal components.
[†]Imputed genotypes

e-Figure 1. Local association plots for significant loci for the meta-analysis of COPD subjects with chronic bronchitis (CB) *versus* COPD subjects without CB in COPDGene non-Hispanic whites and GenKOLS. The x-axis is chromosomal position, and the y-axis shows the $-\log_{10} P$ value. The most significant SNP at each locus is labeled in purple, with other SNPs colored by degree of linkage disequilibrium (r^2). Plots created using LocusZoom.

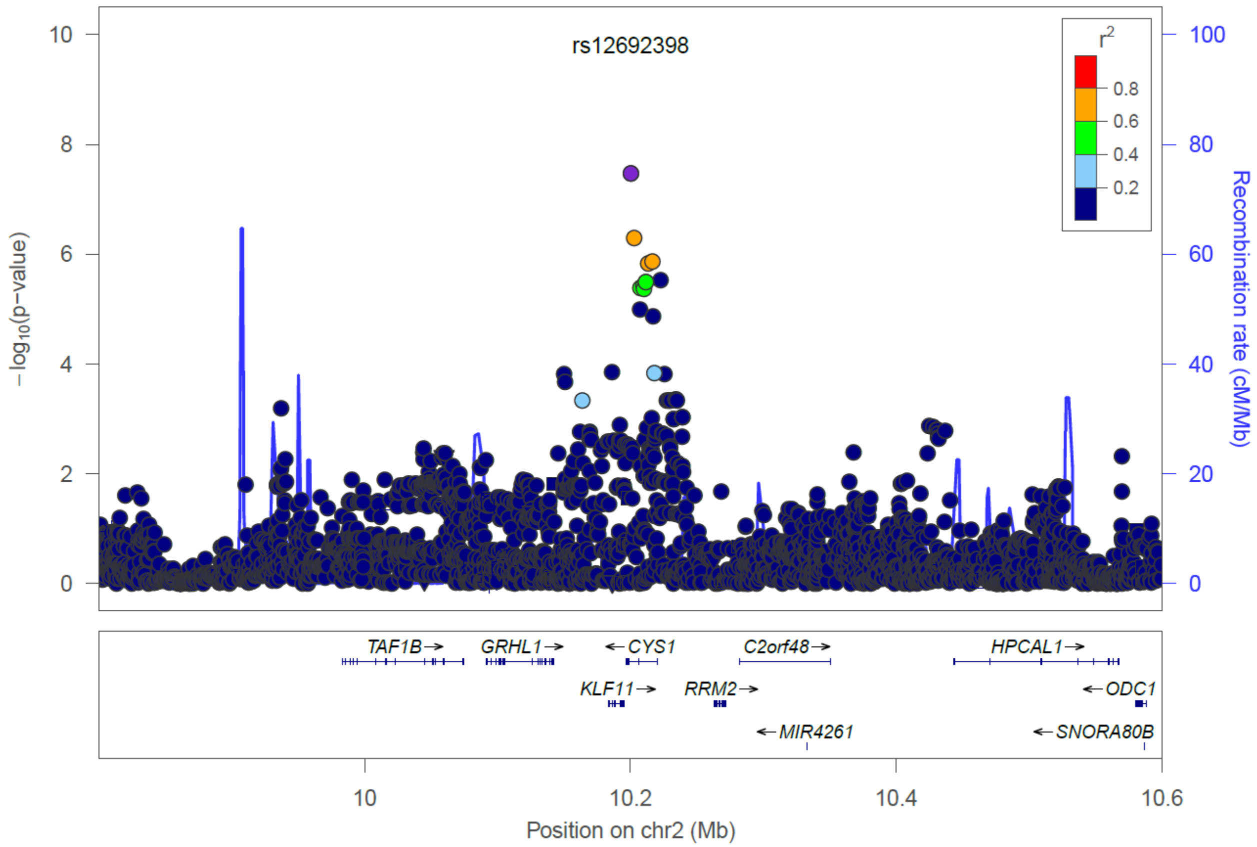

e-Figure 2. (A) The quantile–quantile plot and (B) Manhattan plot of $–\log_{10} P$ values for the three-cohort meta-analysis including 1000 Genomes project imputed data for (A) COPD subjects without chronic bronchitis (CB) *versus* smoking controls after adjustment for age, sex, pack-years of cigarette smoking and genetic ancestry using principal components.

(A) (B)

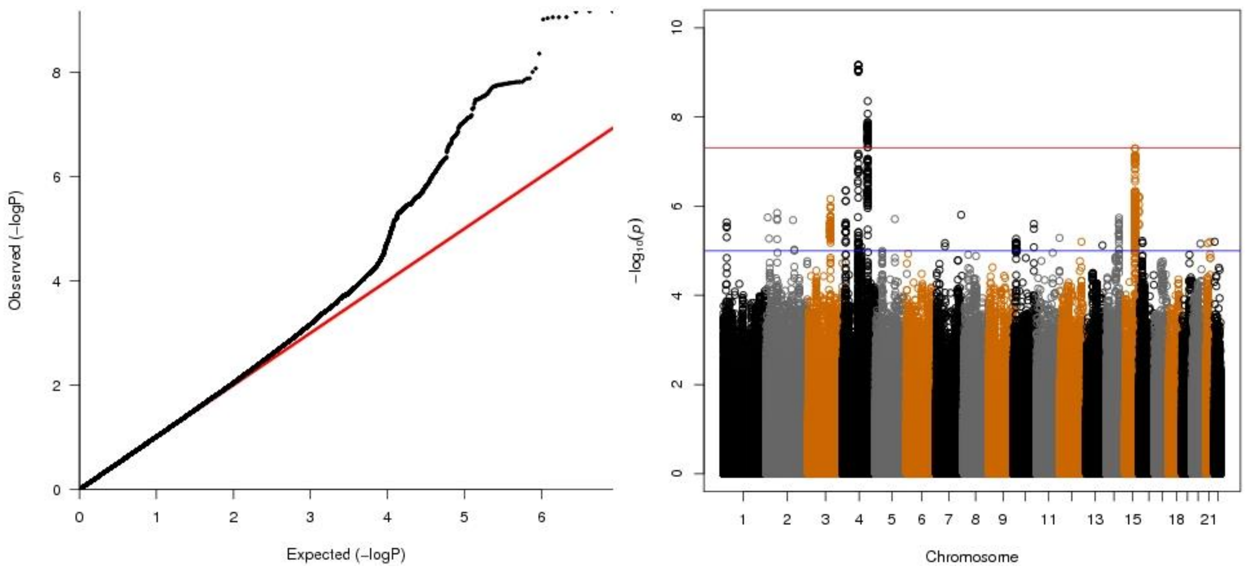

Supplement: Additional file 1: — Variable definitions. Additional analysis methods. Table S1. Top results of the meta-analysis for COPD subjects with chronic bronchitis (CB) versus COPD subjects without CB in COPDGene non-Hispanic white and GenKOLS cohorts. Table S2. Baseline characteristics of COPD subjects with CB and smokers with normal spirometry as a control group in African Americans of COPDGene cohort. Table S3. Baseline characteristics of COPD subjects with CB and smoking controls without CB. Table S4. Top results of the two meta-analyses for COPD subjects with CB versus smokers with normal spirometry, including current smoking adjustment. Table S5. Top results of three Caucasian cohorts meta-analyses for COPD subjects with CB versus without CB, including current smoking adjustment. Table S6. Assessment of top results from COPD with CB versus smokers with normal spirometry within the meta-analysis for COPD subjects with CB versus without CB. Table S7. Clinical and radiological characteristics according to genotypes of rs34391416 among subjects with COPD and smoking controls among non-Hispanic whites of COPDGene. Table S8. Top results of three Caucasian cohorts meta-analyses for COPD subjects without CB versus smokers with normal spirometry. Figure S1. Local association plots for significant loci for the meta-analysis of COPD subjects with CB versus COPD subjects without CB in COPDGene non-Hispanic whites and GenKOLS. The x-axis is chromosomal position, and the y-axis shows the –log10 P value. The most significant SNP at each locus is labeled in purple, with other SNPs colored by degree of linkage disequilibrium (r2). Plots created using LocusZoom. Figure S2. (A) The quantile–quantile plot and (B) Manhattan plot of –log10 P values for the three-cohort meta-analysis including 1000 Genomes project imputed data for (A) COPD subjects without CB versus smoking controls after adjustment for age, sex, pack-years of cigarette smoking and genetic ancestry using principal components. [file 12931_2014_113_MOESM1_ESM.pdf]
